# A Complex Competitive Exclusion Culture Reduces *Campylobacter jejuni* Colonization in Broiler Chickens at Slaughter Age In Vivo

**DOI:** 10.3390/vetsci9040181

**Published:** 2022-04-11

**Authors:** Vanessa Szott, Benjamin Reichelt, Anika Friese, Uwe Roesler

**Affiliations:** Institute for Animal Hygiene and Environmental Health, Freie Universität Berlin, 14163 Berlin, Germany; benjamin.reichelt@fu-berlin.de (B.R.); anika.friese@fu-berlin.de (A.F.); uwe.roesler@fu-berlin.de (U.R.)

**Keywords:** *Campylobacter*, competitive exclusion, CE culture, broiler, control measure, microbiome, intervention strategy

## Abstract

Diminishing *Campylobacter* prevalence in poultry flocks has proven to be extremely challenging. To date, efficacious control measures to reduce *Campylobacter* prevalence are still missing. A potential approach to control *Campylobacter* in modern poultry productions is to occupy its niche in the mucosal layer by administering live intestinal microbiota from adult chickens to dayold-chicks (competitive exclusion (CE)). Therefore, this in vivo study investigates the efficacy of a complex CE culture to reduce *Campylobacter* (*C.*) *jejuni* colonization in broiler chickens. For this purpose, the complex CE culture was applied twice: once by spray application to day-old chicks immediately after hatching (on the 1st day of life) and subsequently by an additional application via drinking water on the 25th day of life. We observed a consistent and statistically significant reduction of *C. jejuni* counts in cloacal swabs throughout the entire fattening period. At the end of the trial after necropsy (at 33 days of age), *C. jejuni* cecal counts also showed a statistically significant decrease of 1 log_10_ MPN/g compared to the control group. Likewise, colon counts were reduced by 2.0 log_10_ MPN/g. These results suggest that CE cultures can be considered a practically relevant control strategy to reduce *C. jejuni* colonization in broiler chickens on poultry farms.

## 1. Introduction

*Campylobacter* is still considered a cause of concern in broiler production as it is the most frequently reported food-borne pathogen in the European Union (EU). In 2019 there were 220,682 confirmed cases of human campylobacteriosis. Although there are many approaches to reduce the burden of *Campylobacter* in poultry, the number of European campylobacteriosis cases in humans remained stable between 2015 and 2019 [1]. Since *Campylobacter* preferentially colonizes the poultry intestinal tract, the elimination in the poultry reservoir must be considered a key step to successfully combat the bacterium in the food chain [2].

In this context, preventing *Campylobacter* contamination in poultry farms remains a major challenge given its ubiquitous occurrence in the environment [3,4,5,6]. On farm, control strategies such as the establishment of well-implemented hygiene protocols have shown to lower the incidence of *Campylobacter* [7,8]. Nevertheless, strict adherence to biosecurity measures does not guarantee that broilers do not become colonized with *Campylobacter* during an entire fattening period [9]. For this reason, it is essential to introduce alternative control strategies to keep *Campylobacter* prevalence in poultry flocks as low as possible [10]. This is because any properly implemented control measure can reduce the likelihood of *Campylobacter* colonization of a poultry flock [11]. So far, effective and applicable measures are still missing or insufficient [10].

A feasible strategy to control *Campylobacter* in poultry flocks is the concept of competitive exclusion (CE). CE is the administration of non-pathogenic intestinal bacteria from adult-chickens to newly hatched-chickens to ensure an early development of mature adult-type microflora that improves animal health and as a result reduces the quantity of pathogenic bacteria [12,13,14]. CE culture treatment has proven effective against *Salmonella* colonization in young chickens [14,15,16] but showed inconsistent effects on *Campylobacter* colonization [17,18,19,20,21,22,23].

A complex mixture of viable commensal bacterial cultures (CE culture) originally isolated from the cecal microbiota of specific pathogen-free adult chickens was used for this study. The CE culture is applicable for chickens and turkeys either as spray treatment immediately after hatch or as drinking water application during the growth phase. The use as a spray aims to increase the broilers’ resistance to subsequent infections caused by harmful bacteria. In addition, when used as a drinking water application after an antibiotic therapy, CE promotes the reestablishment of a balanced microbiota composition in the chicken intestine [12,14]. This second treatment during rearing is expected to boost the effect of CE and consequently contribute to reduced *Campylobacter* colonization [24].

The objective of this study was to determine the efficacy of a complex commercially available CE culture on *Campylobacter* (*C.*) *jejuni* colonization when administered both via spray application and via drinking water to broiler chickens in an in vivo experimental seeder bird model.

## 2. Materials and Methods

### 2.1. Ethics Statement

This study was carried out in accordance with the National Animal Protection Guidelines. The protocol was approved by the German Animal Ethics Committee for the protection of animals of the Regional Office for Health and Social Affairs Berlin (“Landesamt für Gesundheit und Soziales”, LAGeSo, permission number G 0098/18).

### 2.2. Experimental Animal Trial

The study was performed in the experimental facilities of the Centre for Infection Medicine of the Department for Veterinary Medicine of Freie Universität Berlin (biosafety level 2, law on genetic engineering). According to strict, established hygiene management, clothes and shoes were changed in an adjacent separate anteroom before entering the experimental animal facility.

Prior to the beginning of the trials, the experimental units, which had been previously cleaned and disinfected with hydrogen peroxide, were tested for the presence of *Campylobacter* by taking various gauze swabs soaked in sterile phosphate-buffered saline (PBS; Oxoid, Wesel, Germany). Gauze swabs were prepared to perform qualitative *Campylobacter* analysis according to DIN EN ISO 10272-1:2017-09. For this purpose, five selected areas of 10 × 10 cm were individually swabbed. The gauze swabs were transferred to sterile plastic seward stomacher filter bags (Norfolk, UK) containing 20 mL Preston broth (PB) supplemented with Preston *Campylobacter* selective supplement (SR0117; Oxoid, Wesel, Germany), growth supplement (SR0232; Oxoid, Wesel, Germany), and defibrinated horse blood (SR0050; Oxoid, Wesel, Germany). Gauze swabs were homogenized for 2 min at 200 rpm using a stomacher (Seward Stomacher 400 Lab System, Norfolk, UK) and afterwards incubated for 24 h at 37 °C under microaerophilic conditions (85% nitrogen, 10% carbon dioxide, 5% oxygen). One loop material per gauze swab was spread onto modified *Campylobacter*-selective charcoal cefoperazone deoxycholate agar (mCCDA) plates (CM0739; Oxoid, Wesel, Germany) supplemented with CCDA selective supplement (SR0155; Oxoid, Wesel, Germany) using 10 µL inoculation loops (Sarstedt, Nümbrecht, Germany). Plates were incubated for 48 h under microaerophilic atmosphere and examined for *Campylobacter* growth.

For the trials, 180 eggs of broiler breed Ross 308 (both aerosol disinfected with formalin and liquid disinfected with WESSOCLEAN^®^ K 50 Gold Line (Wesso AG, Hersbruck, Germany) were received from a commercial poultry production and incubated for 21 days until hatch. Newly hatched chickens of both sexes were then randomly assigned to two groups of 90 chickens each: either the positive control group (challenged by oral gavage with *Campylobacter* and not treated—T1) or the CE group (challenged by oral gavage with *Campylobacter* and treated with the complex CE culture—T2). In order to imitate a commercial broiler chicken husbandry, broiler chickens were housed in separate units at a stocking density of 39 kg/m^2^ on ground floor with fresh litter (1 kg/m^2^)—litter was neither removed nor added throughout the experimental period. Temperature, filtered air (ventilation and HEPA filtration of the exhaust air), and light were controlled throughout the entire study period and adjusted properly as to the broilers’ age. Broilers had access to drinking water (tap water) and feed *ad libitum.* The chickens received a conventional three-phase diet as shown in Table 1. Fresh water was provided on a daily basis. On the 1st day of life, each chick was randomly tagged with a unique number to distinguish between seeders (orally inoculated with *C. jejuni*, *n* = 18), sentinels (repeatedly sampled non-inoculated contact animals, *n* = 36), and stocking density broilers (non-inoculated and non-sampled contact animals, *n* = 36). At the age of 10 days, the seeders were orally challenged with approximately 10^4^ colony forming units (cfu)/500 µL of *C. jejuni* to assess natural transmission within the broilers, as *C. jejuni* will spread from the seeders to the non-infected contact animals. Consequently, the contact animals were naturally colonized with *C. jejuni*. For the determination of *C. jejuni* colonization, seeders and sentinels were sampled by means of cloacal swabs. Health and weight gain of the animals were supervised and documented daily. At the end of the trial, at 33 days of age (average weight 2.0 kg), animals were euthanized, dissected and *Campylobacter* counts were determined in the cecal and colon contents of the sentinels.

### 2.3. Application of the Complex Competitive Exclusion Product

The product used in the study was the competitive exclusion product Aviguard^®^ (Lallemand, Worcestershire, UK). The compound contains the following bacterial species (approximately 10^9^ cells per g, as specified by the manufacturer): *Escherichia coli*, *Citrobacter species*, *Enterococcus species* (*E. faecalis*, *E. faecium*), *Lactobacillus species* (*L. casei*, *L. plantarum*), *Bacteroides species*, *Clostridium species* (*C. sporogenes*), *Eubacterium species*, *Propionibacterium species*, *Fusobacterium species*, *Ruminococcus species* [25]. To examine the efficacy of the CE culture, broilers in the T2 group received the product twice: (i) via spray application and (ii) via drinking water according to manufacturer’s specifications. Simultaneously with each administration, the CE culture was examined for the presence of *Campylobacter.* In brief, the CE culture suspensions were homogenized in sterile PBS (Oxoid, Wesel, Germany) and assayed in serial dilutions plated in 100-µL aliquots on mCCDA plates. The absence of *C. jejuni* growth was detected after the bacteria were grown for 48 h at 37 °C in a microaerophilic atmosphere.

The spray application was performed immediately after hatching. For this purpose, about 20 min before treatment, the entire sachet of the CE culture (25 g) was dissolved in 500 mL (amount for 2000 chickens) of tap water. From this homogenized total volume, 25 mL CE culture solution (amount for one hundred chickens) was taken and poured into three manual sprayer devices delivering coarse droplets (for 30 chickens each). We used the amount for one hundred chickens since there is always some residue in the spray bottle. In total, each chicken in the T2 group was treated with 0.25 mL of the prepared CE culture solution. To ensure an accurate application during spraying procedure and thus allow sufficient CE culture uptake for an early mature colonization, chickens were divided among three plastic boxes (each containing 30 chickens) and then treated simultaneously with the CE culture. After a short application time (five to ten minutes), broiler chickens were allocated to their corresponding pen. The administration via drinking water was performed one week before necropsy (at 25 days of age). In order to treat 90 chickens, of the total amount of 25 g (amount for 2000 chickens), 1.125 g of CE culture was dissolved in 1 L of tap water, thoroughly homogenized and then added to 8 L of drinking water (corresponding to the water consumption of 90 broiler chickens). The CE culture was provided to the broilers via nipple drinkers for six hours. Within this treatment period, the entire amount of water was consumed by the chickens. The drinking buckets were thoroughly rinsed out and refilled with fresh tap water.

### 2.4. C. jejuni Strain and Seeder Inoculation

Oral inoculation of the seeders was conducted using a comprehensively characterized *C. jejuni* reference strain BfR-CA-14430 (characterized by whole genome sequencing (WGS) and multilocus sequence typing (MLST), which was originally isolated from poultry (chicken breast). This particular strain belongs to the MLST clonal complex (CC)21, which on the one hand is one of the largest clonal complexes found so far [26] and on the other hand is highly prevalent in livestock and different environmental sources worldwide [27,28]. Indeed, *C. jejuni* genomes associated with CC21 are often found in chickens but also met the criteria for host-generalist lineages [29]. In addition, it is frequently associated with human disease cases [30].

For the experiments, an inoculum containing 3.4 × 10^4^ cfu of *C. jejuni* was prepared and analyzed as described earlier [31]. Seeders were then orally inoculated individually into the crop with 0.5 mL of the prepared bacterial suspension.

### 2.5. Sampling Design and Microbiological Analysis

Prior to oral inoculation, all broilers were monitored for the presence of *Campylobacter* by taking cloacal swabs (Sarstedt, Nümbrecht, Germany) at four days of age.

Throughout the study, *C. jejuni* colonization of broilers was determined by semi-quantitative analysis of cloacal swabs. Cloacal swabs were collected in a standardized manner (time of sample collection, method of sample collection, sample processing). Cloacal swabs were taken as follows: on three consecutive days (namely 2, 3 and 4 days post inoculation (dpi)) (corresponds to day 12, 13, and 14 of age), then at least twice a week (8, 11, 16, and 18 dpi) (equivalent to day 18, 21, 26, and 28 of age). To ensure comparability of results, the same 36 sentinels (non-inoculated, but naturally colonized with *C. jejuni* through contact with the seeders) were sampled in both groups. Seeders were examined only once for *C. jejuni* excretion by collection of cloacal swabs 2 dpi.

Cloacal swabs were analyzed semi-quantitatively and adapted to International Organization for Standardization/Technical Specifications (ISO/TS) 10272-3:2010. Cloacal swabs were inserted in the cloaca, rotated five times, removed and immediately transferred to 3.0 mL PB. Thereafter, cloacal swabs were homogenized for three seconds using a vortex shaker (VWR. Darmstadt, Germany), allowing the fecal material to detach and evenly disperse in the medium. Afterward, cloacal swabs were 10-fold serially diluted in PB (up to 10^−8^) and bacteria were grown for 24 h at 37 °C in a microaerophilic atmosphere. Dilutions were then streaked onto quartered mCCDA plates using 10 µL inoculation loops (Sarstedt, Nümbrecht, Germany). Plates were incubated for another 48 h under the same conditions and examined for *C*. *jejuni* growth. The highest dilution with confirmed *Campylobacter* growth was used to determine the MPN (Most Probable Number) value. The result was determined using an MPN table modified according to ISO/TS 10272-3:2010/Cor.1:2011(E).

At 33 days of age (average weight 2.0 kg), all 36 sentinels per group were euthanized using ZKS poultry pliers (Corstechnology, Neerstedt, The Netherlands) after confirming deep anesthesia. The animals were dissected and intestinal contents (from cecum and colon) were collected for subsequent *C. jejuni* enumeration. Necropsy samples were prepared to perform semi-quantitative analysis also adapted to ISO/TS 10272-3:2010. Approximately 1 g of gut content was removed aseptically, diluted 1:8 in PB, thoroughly homogenized, and then ten-fold diluted in PB to 1 × 10^−9^. Diluted intestinal samples were then processed as described above. After incubation for 48 h at 37 °C under microaerobic atmosphere, the highest dilution showing bacterial growth was assessed.

### 2.6. Statistical Analysis

Statistical analysis was carried out using SPSS software version 25.0 for Windows (SPSS, Inc., Chicago, IL, USA). Before statistical analysis, individual *Campylobacter* counts were transformed to log_10_ counts and then used as the experimental unit. The Shapiro–Wilk test was used to test the normal distribution of the data. Since our data did not meet criteria of normal distribution, we applied pairwise comparisons using the non-parametric Mann–Whitney *U*-test. To ensure alpha error of 0.05, β-error of 0.18 and power of 0.80, 90 animals per group were included in the present study. In order to determine statistically significant differences, 36 animals were sampled during the animal trial. Probability (*p*)-values below 0.05 were considered statistically significant.

## 3. Results

### 3.1. CE Culture

No *Campylobacter* spp. was cultivated from the CE culture used.

### 3.2. Effect on Colonization

**Figure 1:**
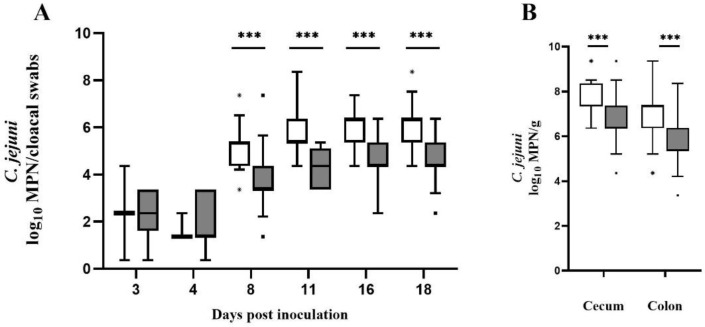
*C. jejuni* colonization of 36 sentinels per group determined by semi-quantitative analysis. *C. jejuni* counts in log_10_ most probable number (MPN) of 36 sentinels per group (**A**) in cloacal swabs at distinct time points after oral inoculation of the seeders on day 10 and (**B**) per gram in intestinal content upon necropsy (day 23 post inoculation). White boxes feature the control group (broilers challenged with *C. jejuni* and not treated with the CE culture); gray boxes feature broilers challenged with *C. jejuni* and treated with the CE culture on days 1 (via spray) and 25 (via the drinking water). The box plots show the 5th and 95th percentiles (whiskers) and outliers (shown as asterisk (*) for the control group and black square for the CE group). Medians (bold line) and significance levels (*p* values) determined by the Mann Whitney *U*-test are indicated. Time points showing a significant reduction (*p* < 0.0001) in *Campylobacter* counts compared to the control group are marked with three asterisks.

The semi-quantitative results of both groups are presented in Figure 1. Prior to oral inoculation, *C. jejuni* was not detectable in any of the broilers. All seeders per group shed *C. jejuni* 2 dpi. The determined *Campylobacter* counts from cloacal swabs collected from seeder birds were similar in both groups (*Md* = 1.86 log_10_ MPN/cloacal swabs in the control group vs. *Md* = 2.36 log_10_ MPN/cloacal swabs in the CE group). Initial sampling of sentinels (3 dpi) showed that although the number of positive *Campylobacter* excretors in the control group was comparatively lower (2 sentinels in the control group vs. 8 sentinels in the CE group), the *C. jejuni* counts obtained from cloacal swabs were the same (*Md* = 2.36 log_10_ MPN/cloacal swabs). Likewise, 4 dpi fewer sentinels (*n* = 3) in the control group excreted *C. jejuni* than in the CE group (*n* = 21). *Campylobacter* counts determined 4 dpi from cloacal swabs were slightly lower in the control group (*Md* = 1.36 log_10_ MPN/cloacal swabs) than in the CE group (*Md* = 1.72 log_10_ MPN/cloacal swabs). All sampled sentinels were positive for *C. jejuni* 8 dpi. Comparing both groups, *Campylobacter* counts in cloacal swabs were significantly and consistently lower (*p* < 0.0001) in the CE group at 8, 11, 16, and 18 dpi (Figure 1A). At 8, 16, and 18 dpi, cloacal swabs from the CE group demonstrated the highest decrease in *C. jejuni* counts (*Md* = 3.36, 4.36, and 4.36 log_10_ MPN/cloacal swabs) in comparison to the control group (*Md* = 5.36, 6.36 and 6.36 log_10_ MPN/cloacal swabs). These results correspond to a log reduction of 2 log_10_ MPN/cloacal swabs (at 8 dpi *p* < 0.0001; *r* = 0.67, at 16 dpi *p* < 0.0001; *r* = 0.63 and at 18 dpi *p* < 0.0001; *r* = 0.59) respectively. Similar results could be observed at 11 dpi. Sentinels treated with the CE culture revealed to have significantly lower *Campylobacter* counts (*Md* = 4.36 log_10_ MPN/cloacal swabs) compared to the control group (*Md* = 5.36 log_10_ MPN/cloacal swabs), corresponding to a log reduction of 1 log_10_ MPN/cloacal swabs (*p* < 0.0001; *r* = 0.68). Furthermore, the analysis of cecal samples demonstrated a significant decrease (*p* < 0.0001; *r* = 0.46) of *Campylobacter* cecal colonization (Figure 1B) for the CE group (*Md* = 6.36 log_10_ MPN/g) compared to the control group (*Md* = 7.36 log_10_ MPN/g). The observed log reduction in *Campylobacter* cecal counts for the CE group was 1 log_10_ MPN/g. Equally, *C. jejuni* counts in the colon of CE culture treated broilers were significantly reduced (*Md* = 5.36 log_10_ MPN/g) (*p* < 0.0001; *r* = 0.45) in comparison to the control group (*Md* = 7.36 log_10_ MPN/g). The log reduction in *Campylobacter* colon counts of sentinels receiving the CE culture was 2 log_10_ MPN/g (Figure 1B). Individual *C. jejuni* counts of seeder and sentinels are presented in Tables S3 and S4.

### 3.3. Effect on Broilers’ Performance

CE culture treatment showed no effect on the animals’ growth performance as presented in Tables S1 and S2. At the end of the trial, no significant differences in the final mean body weight were observed (*p* > 0.05) between sentinels of the experimental (1.98 kg) and sentinels of the control group (1.87 kg).

## 4. Discussion

Supporting the development of a mature and competitive microbiota by administering intestinal content of adult birds to newly hatched chickens is a promising approach to reduce *Campylobacter* cecal colonization. Previous research has indicated a profound mutual interdependence between *Campylobacter* and the present ubiquitous microbiome. On the one hand, the ability of *Campylobacter* to colonize the intestinal cecal crypts was influenceable by cecal microbiota composition. On the other hand, previous research demonstrated that *Campylobacter* colonization itself induces a shift in the intestinal microbiome, especially the beta-diversity (the variability in community composition within the same habitat) [32,33].

In this in vivo study, we examined whether a complex CE culture has the potential to reduce *Campylobacter* carriage in broiler chickens at slaughter age when administered via spray application and via drinking water. As far as we are aware, this is the first in vivo study to evaluate the efficacy of this complex CE culture in reducing *Campylobacter* colonization in broiler chickens using a practical setup that approximates commercial poultry farming, as two administration methods common to conventional poultry operations were used. This approach differs from earlier in vivo attempts, in which CE cultures were mostly administered via an oral gavage into the broilers’ crop [24,34,35,36,37] in order to ensure an accurate dosage per chicken [14]. However, these experimental setups are difficult to implement on poultry farms and do not conform to current administration methods. Therefore, especially regarding practicability, we chose a simpler experimental approach which has been shown to be as effective as direct gavage into broilers’ crop [38]. This method was first introduced by Pivnick and Nurmi [39] and can be easily repeated in field studies or on poultry farms without subjecting the broilers to any undue strain or stress. In particular, spray application with coarse droplets is a proven method ensuring a quick ingestion because sparkling spray droplets on the feathers excite the day-old chickens to preen themselves [14] while being harmless and without any adverse effects [14,40]. Besides, an early administration of CE cultures shortly after hatch is advisable to rapidly induce the formation of a yet stable gut microbiota [41], as chicken cecal microbiota becomes diverse and stable with increasing age [42]. In addition, we administered the CE culture via broilers’ drinking water, as this is a common administration method in commercial broiler production [43].

The results of this in vivo study are encouraging as the CE culture reduced *C. jejuni* load in cloacal swabs significantly and consistently throughout the fattening period (the maximum observed log reduction was 2 log_10_ MPN). Moreover, at the end of the trial after necropsy we determined a significantly decreased *C. jejuni* cecal load in 33-day-old broilers. In comparison to the control group, the cecum of broilers receiving the CE culture showed significantly reduced *Campylobacter* counts (log reduction of 1 log_10_ MPN/g). Likewise, colon counts were significantly lower (log reduction of about 2 log_10_ MPN/g). Based on the relationship between *Campylobacter* concentrations in the ceca and corresponding broiler carcass skin samples, a 2-log_10_ reduction in broiler cecal concentrations is estimated to reduce the relative risk of human campylobacteriosis in the EU by 42%, while a 3-log_10_ reduction in broiler cecal concentrations would reduce the risk by as much as 58% [11]. Although the effect of the CE culture on *Campylobacter* cecal colonization was modest (1 log_10_ MPN/g), it is important to note that any reduction in *Campylobacter* numbers in the cecal content may contribute to reduce the *Campylobacter* load on the broiler carcasses during processing [44,45].

One may argue that (i) cloacal swabs are an unreliable source for quantitative *Campylobacter* detection (varying or low amounts of feces adhering to the swab) and (ii) quantitative analysis of samples may have been more accurate. Indeed, quantitative analysis of samples where high *Campylobacter* counts are expected is considered the gold standard for the detection and quantification of *Campylobacter* and determination of *Campylobacter* concentrations via cloacal swabs is not the most reliable method available. However, selective sampling of sentinels is required for analysis of natural infection models such as those used in the present study. Indeed, *C. jejuni* enumeration of fecal samples, or in particular cecal droppings might have been more accurate to illustrate the cecal *Campylobacter* colonization of the broilers. However, the collection of cecal droppings of certain sentinels (as necessary in this study) was not feasible in our experimental setting for the following reasons: (i) broiler chickens excrete them infrequently [46] and (ii) the isolation of seeder and sentinel broilers for a prolonged period of time would have compromised the experimental seeder bird model, which targets natural infection and keeps conditions close to commercial poultry production. The use of cloacal swab ensured the sampling of “naturally” infected sentinels and thus the examination of the individual course of each of the 36 sentinels (by assigning samples to the tag number). Furthermore, this allowed us not only to include a large sample size in our study but also to assess *Campylobacter* reduction under real-life conditions. To address the varying amounts of feces adhering to the swab and the associated difficulties in quantification, we used the semi-quantitative method and a standardized sampling and sample processing procedure to obtain comparable and reproducible data. The reproducibility and accuracy of the data of the present approach are satisfactory, as in the control group the *Campylobacter* counts in cloacal swabs were consistently homogeneous regardless of the time of sampling (11, 16, and 18 days post inoculation). In line, a previous study showed a similar isolation rate between direct culture on mCCDA and enrichment when pooled cecal samples from different slaughter batches were examined [47]. Likewise, another study found no statistical difference between enumeration by the semi-quantitative and quantitative technique for comparable concentrations of thermotolerant *Campylobacter* (*p* = 0.104) [48]. Similarly, no significant differences (*p* > 0.05) could be detected between results obtained by direct plating of carcass rinse samples on Campy-cefex agar and an MPN procedure [49]. In support, Scherer and colleagues observed a highly positive correlation coefficient of 0.9 between direct plating and MPN technique and therefore concluded both methods to be suitable for the detection and quantification of *Campylobacter* [50].

Although our results are auspicious, earlier attempts using CE cultures of different compositions showed varying potential to lower *Campylobacter* colonization [14,17,18,19,21,22]. Stern [51] found no reducing effect of a conventional CE preparation on *Campylobacter* colonization. A preparation made from cecal wall material (MCE culture), however, yielded lower *Campylobacter* cecal colonization (average reduction 2.01 log cfu/g cecal material) [35]. In addition, Hakkinen and Schneitz [34] displayed the efficacy of another commercially available CE product Broilact^®^ (Orion Corporation, Espoo, Finland) against *Campylobacter jejuni* colonization in Ross I broiler chickens 12 days after oral challenge. Consistent with the results of the present study, Ty and colleagues [52] observed reduced *C. jejuni* colonization in Ross 708 broilers 39 days post hatching, following a single administration of Aviguard^®^ via drinking water on the first day of life. To increase the protective effect of CE cultures, Mead et al. [14] contemplated that an extended time period between CE treatment and challenge might be beneficial which is why in this approach we defined a 10-day interval between CE culture administration and artificial *C. jejuni* challenge. Additionally, CE culture retreatment during rearing might boost the CE effect [24]. In accordance, Schoeni et al. [19] demonstrated the advantageous effect of an additional booster treatment on *Campylobacter* colonization in White Leghorn cockerel chickens. Accordingly, in this study we observed a consistent and significant reduction of *C. jejuni* in cloacal swabs after CE booster treatment, as both cloacal swabs taken 1 and 3 days after CE booster treatment (corresponding to 16 and 18 dpi) revealed lower *C. jejuni* counts (log reduction of 2 log_10_ MPN/cloacal swabs) as shown for the control group. Based on these observations in conjunction with the results after necropsy, it can be speculated that a CE booster treatment might contribute to reducing *C. jejuni* colonization. Whether the second treatment had a protective effect remains to be determined. In addition, the colonization of broilers with *C. jejuni* may naturally be subject to individual variation, and the present study cannot fully clarify whether or to what extent these natural variations may have affected our results.

With regard to the use of CE cultures, several other factors might affect their efficacy, namely rearing conditions, administration as well as challenge method, time of administration, CE culture composition (few bacterial strains or abundance of different bacteria), donor material, bird strain, stress, and rearing length [14,39,53]. Moreover, it should be considered that the composition of CE cultures may vary considerably between different batches. Reasons for this variability are that several parameters such as environmental (seasonal and geographical climate changes), host factors (genetics, gut development), increasing broiler age [54], hygiene, medication, housing and switch of feed type (starter, grower, finisher) may contribute to changes in microbiota abundance and diversity in donor chickens [42,55,56]. Consequently, the efficacy of the CE culture may be compromised if the number of bacterial strains that contribute significantly to CE activity is reduced [57]. Nevertheless, the content of the CE culture was not investigated in the present study, so we cannot be certain whether this product itself or only the content of this batch has an effect. This should be explored and needs to be verified in further studies.

It appears that commercially available CE products are effective due to the complexity and richness of naturally occurring elements of the normal microbiota [14] on the one hand, and the presence of facultative anaerobic bacteria on the other hand. Nevertheless, it should be mentioned that recent research has aimed at identifying potential defined CE cultures (consisting of a small proportion of well-characterized bacteria) that show similar potential against *Campylobacter* colonization as has been observed for commercially available CE products. However, those studies yielded different competitive strains and outcomes [17,18,19,21,58].

Notwithstanding the efficacy of complex CE products, they have not yet been approved for poultry fattening in Germany. As early as in 1994, the World Health Organization (WHO) suggested to classify CE products as “normal gut flora”, a classification provided for license simplification, since they do not fall precisely in one of the following categories: vaccine, feed additive, or veterinary medicinal product [59]. Indeed, due to their complex nature, the establishment of an appropriate regulatory framework for the placing on the market of CE cultures appears to be quite challenging. Many species of the intestinal microbiota have not yet been fully identified [57] nor have they ever been cultivated [60]. Despite the current scientific progress, it is still difficult to achieve a complete characterization of their contents as required by some legislation, for example the European Regulation on additives for use in animal nutrition (Regulation (EC) No 1831/2003). Moreover, CE cultures may be considered a potential source of pathogens [14] and may harbor transferable antimicrobial drug resistance or virulence genes [57] that could pose a risk to human health. Although a few European countries have their own framework which allows the approval of CE products, a harmonized EU regulatory framework is still missing [61]. In fact, in many countries around the world CE cultures are registered under national legislation either as veterinary medicines or as feed additives/probiotics. CE cultures have been and are still being used successfully in several European countries (e.g., UK, Sweden, Norway and Finland). Notably, the low incidence of *Campylobacter* in Finnish broiler flocks has been indicated to correlate with the consistent long-term use of CE cultures against *Salmonella* [24,62].

## 5. Conclusions

In conclusion, CE cultures can be considered a valuable concept for the control of *Campylobacter* on poultry farms although the entire mode of action is not yet clearly elucidated. At present, it is difficult to assess the extent to which CE cultures are effective and which specific factors are responsible for their effectiveness. As observed previously, it appears that both defined and complex CE products may diminish *Campylobacter* colonization in broiler chickens. The results of the present study are encouraging and can be considered of practical relevance in poultry production as conventional administration of the CE culture significantly reduced *Campylobacter* cecal colonization in broilers at slaughter age. However, in Germany full disclosure of the composition of CE products are required for authorization to ensure health safety of human consumers. For this reason, enlightening endeavors are needed to comply with this requirement. Since the chickens in this study were raised under favorable experimental conditions, it is also necessary to verify whether the results obtained can be reproduced with larger broiler flocks under commercial conditions. In order to draw careful conclusions, further studies are needed that advance microbiome analyses, especially through innovative and sophisticated sequence techniques. These outstanding tools are essential to gain important insights into the interplay between *Campylobacter* and the currently ubiquitous microbiome to adequately examine the suitability of CE cultures for commercial poultry productions. In addition, it should be investigated whether a reduction in *C. jejuni* colonization can be achieved by only one of the two CE treatments or whether the second application in the drinking water can actually contribute to a reduction in *C. jejuni* counts (increased protection). Furthermore, the simultaneous combination of CE cultures and other control strategies may be a promising approach for further reducing *Campylobacter* colonization in broiler chickens.

## Figures and Tables

**Table 1:** Composition and analytical constituents of the experimental three-phase diet.

Ingredients, per kg	Starter Feed (Day 0–8)	Grower Feed (Day 9–26)	Finisher Feed (Day 27–33)
Crude protein (%)	21.5	21.0	20.0
Crude lipids (%)	4.9	6.4	5.5
Crude fiber (%)	2.9	3.4	3.3
Crude ash (%)	5.3	5.1	4.9
MJ ME ^1^	12.4	12.4	12.4
Calcium (%)	0.9	0.8	0.8
Phosphorous (%)	0.6	0.55	0.5
Sodium (%)	0.14	0.14	0.14
Methionine (%)	0.55	0.50	0.50
Lysine (%)	1.25	1.15	1.05

^1^ megajoules of metabolizable energy.

## Data Availability

The data presented in this study are contained within the article and available in the Supplementary Materials.

## Supplementary Materials

The following supporting information can be downloaded at: https://www.mdpi.com/article/10.3390/vetsci9040181/s1. Table S1: Mean body weight (g) of broiler chickens in the control group during the animal trial. Ten randomized broiler chickens were weighed daily. Table S2: Mean body weight (g) of broiler chickens treated with the CE culture during the animal trial. Ten randomized broiler chickens were weighed daily. Table S3: C. jejuni counts (log10 MPN) of seeder and sentinel broiler chickens at 2, 3, 4, 8, 11, 16, and 18 days after inoculation from cloacal swabs. Table S4: C. jejuni cecal and colon colonization of sentinel broiler chickens at 33 days of age after necropsy.
