# Supplementary material for: A Complex Competitive Exclusion Culture Reduces Campylobacter jejuni Colonization in Broiler Chickens at Slaughter Age In Vivo
Source: Vet Sci. 2022 Apr 11;9(4):181. doi: 10.3390/vetsci9040181 (PMC9029414; doi:10.3390/vetsci9040181)
Supplement: Supplementary file 1 [file vetsci-09-00181-s001.zip › Supplementary table S1_2.pdf]

Supplementary Table S1. Mean body weight (g) of broiler chickens in the control group during the animal trial. Ten randomized broiler chickens were weighed daily.

	Day																	
	**d1**	**d2**	**d3**	**d4**	**d5**	**d6**	**d7**	**d8**	**d9**	**d10**	**d11**	**d12**	**D13**	**d14**	**d15**	**d16**	**[1]d 33 Necropsy**	
	36	36	56	51	77	92	126	97	166	203	240	187	224	233	354	376	1765	1120
	37	49	60	53	81	107	126	102	175	210	264	198	294	242	395	456	1780	1460
	38	49	62	69	82	114	126	125	197	221	266	203	300	270	410	474	1800	1660
	38	50	63	72	90	118	132	137	198	244	278	214	316	339	412	485	1800	1700
	39	51	65	75	92	120	135	149	199	248	283	242	330	353	421	494	1800	1742
	39	~~52~~	65	80	93	122	141	163	199	249	284	242	335	354	430	505	1852	1757
	40	55	66	80	96	127	143	170	205	260	292	249	339	355	442	515	1855	1764
	40	55	68	82	106	129	148	172	216	260	294	263	341	380	475	542	1880	1800
	40	58	69	84	107	130	152	172	219	263	298	267	350	393	492	610	1886	1821
	40	60	71	84	109	132	156	200	222	284	320	268	360	400	550	615	1907	1861
**BW (g)**	**38.7**	**51.5**	**64.5**	**73.0**	**93.3**	**119.1**	**138.5**	**148.7**	**199.6**	**244.2**	**281.9**	**233.3**	**318.9**	**331.9**	**438.1**	**507.2**	1926	1876
																	1930	1880
	**d17**	**d18**	**d19**	**d20**	**d21**	**d22**	**d23**	**d24**	**d25**	**d26**	**d27**	**d28**	**d29**	**d30**	**d31**	**d32**	1944	1893
	363	394	608	569	482	812	746	909	658	712	744	820	859	910	1566	1672	1987	1924
	484	402	616	575	714	825	859	931	755	862	879	1000	1086	1160	1659	1708	2150	1925
	501	430	645	710	725	884	891	1015	915	979	1146	1188	1288	1341	1686	1730	2179	1980
	515	460	680	759	745	890	900	1045	1033	979	1157	1208	1305	1449	1721	1853	2200	2089
	518	512	698	762	790	920	987	1060	1132	1060	1230	1232	1452	1470	1777	1921	2217	2125
	540	513	702	765	800	930	1033	1065	1138	1089	1255	1244	1560	1484	1795	1960		
	543	527	715	782	812	964	1060	1233	1155	1100	1264	1280	1570	1753	1799	2008		
	587	533	810	797	825	1052	1065	1297	1200	1130	1273	1300	1580	1784	1854	2021		
	596	541	858	810	850	1124	1202	1336	1206	1160	1313	1300	1580	1798	1865	2084		
	611	552	885	919	860	922	1237	1245	1207	1160	1414	1300	1607	1834	2057	2105		
**BW (g)**	**525.8**	**486.4**	**721.7**	**744.8**	**760.3**	**932.3**	**998**	**1113.6**	**1039.9**	**1023.1**	**1167.5**	**1187.2**	**1388.7**	**1498.3**	**1777.9**	**1906.2**	**1867.6**	

[1]The weights on the day of necropsy were obtained from the 36 sentinels examined

Supplementary Table S2. Mean body weight (g) of broiler chickens treated with the CE culture during the animal trial. Ten randomized broiler chickens were weighed daily.

	day																	
	**d1**	**d2**	**d3**	**d4**	**d5**	**d6**	**d7**	**d8**	**d9**	**d10**	**d11**	**d12**	**d13**	**d14**	**d15**	**d16**	**[1]d33 Necropsy**	
	59	56	62	107	129	166	193	224	280	301	379	425	497	512	568	603	1523	1954
	60	56	69	104	133	174	198	237	298	340	405	433	520	516	617	638	1665	1977
	60	57	71	104	120	147	190	222	275	300	359	415	486	511	559	597	1772	2049
	61	58	75	104	117	146	177	213	260	293	351	413	476	489	542	581	1777	2055
	61	61	76	102	116	139	174	209	255	278	348	397	447	486	532	548	1795	2066
	61	62	78	101	115	137	170	202	238	262	347	383	438	468	501	539	1798	2068
	62	64	80	100	114	133	169	199	210	259	309	380	410	447	490	532	1802	2077
	63	65	81	100	110	129	159	177	201	249	269	374	371	435	479	502	1818	2112
	64	67	82	100	101	126	156	173	198	236	258	364	362	433	477	500	1818	2113
	64	73	82	97	100	123	150	168	188	209	242	355	349	401	469	441	1855	2132
**BW (g)**	**61.5**	**61.9**	**75.6**	**101.9**	**115.5**	**142**	**173.6**	**202.4**	**240.3**	**272.7**	**326.7**	**393.9**	**435.6**	**469.8**	**523.4**	**548.1**	1878	2141
																	1885	2159
	**d17**	**d18**	**d19**	**d20**	**d21**	**d22**	**d23**	**d24**	**d25**	**d26**	**d27**	**d28**	**d29**	**d30**	**d31**	**d32**	1892	2171
	560	547	653	667	892	786	866	1074	1037	1101	1360	1298	1546	1380	1510	1523	1894	2179
	596	605	662	698	898	957	1018	1160	1060	1190	1400	1430	1565	1418	1730	1665	1895	2186
	614	618	700	730	906	1033	1022	1160	1168	1213	1400	1502	1588	1640	1782	1772	1907	2206
	680	633	707	812	918	1051	1026	1172	1315	1245	1400	1515	1699	1649	1819	1777	1916	2313
	681	648	722	887	953	1061	1042	1200	1333	1250	1400	1520	1709	1699	1867	1795	1927	2775
	702	734	740	919	988	1067	1114	1215	1359	1350	1407	1533	1730	1840	1879	1798		
	709	755	750	960	998	1105	1196	1220	1375	1379	1408	1544	1732	1850	1987	1802		
	774	810	764	966	1107	1205	1342	1400	1521	1530	1964	1685	2100	2239	2325	1855		
	730	765	798	994	999	1118	1206	1290	1453	1408	1600	1601	1742	1920	1992	1818		
	737	795	805	1000	1041	1152	1339	1315	1513	1490	1620	1624	1876	1980	2064	1818		
**BW (g)**	**678.3**	**691**	**730.1**	**863.3**	**970**	**1053.5**	**1117.1**	**1220.6**	**1313.4**	**1315.6**	**1495.9**	**1525.2**	**1728.7**	**1761.5**	**1895.5**	**1762.3**	**1987.5**	

[1]The weights on the day of the necropsy are from the 36 sentinels examined
