# Supplementary material for: A Complex Competitive Exclusion Culture Reduces Campylobacter jejuni Colonization in Broiler Chickens at Slaughter Age In Vivo
Source: Vet Sci. 2022 Apr 11;9(4):181. doi: 10.3390/vetsci9040181 (PMC9029414; doi:10.3390/vetsci9040181)
Supplement: Supplementary file 1 [file vetsci-09-00181-s001.zip › Supplementary table S3.pdf]

Supplementary Table S3. *C. jejuni* counts (log_{10} MPN) of seeder and sentinel broiler chickens at 2, 3, 4, 8, 11, 16 and 18 days after inoculation from cloacal swabs.

	[1]2 d post inoculation		[2]3 d post inoculation		[2]4 d post inoculation		[2]8 d post inoculation		[2]11 d post inoculation		[2]16 d post inoculation		[2]18 d post inoculation	
	**control**	**CE-culture**	**control**	**CE-culture**	**control**	**CE-culture**	**control**	**CE-culture**	**control**	**CE-culture**	**control**	**CE-culture**	**control**	**CE-culture**
	0.36	2.36	nd	nd	nd	1.36	4.36	4.36	5.36	3.36	6.36	4.36	7.36	3.36
	3.36	1.36	nd	nd	nd	nd	5.36	3.36	5.36	3.36	6.36	6.36	6.36	6.36
	2.36	1.36	nd	nd	nd	nd	5.36	3.36	6.36	4.36	4.36	3.36	6.36	4.36
	0.36	2.36	nd	nd	2.36	nd	5.36	4.36	7.36	5.36	6.36	5.36	8.36	6.36
	1.36	2.36	nd	nd	nd	1.36	4.36	3.36	5.36	3.36	5.36	2.36	5.36	4.36
	1.36	1.36	nd	1.36	nd	3.36	5.36	4.36	5.36	5.36	5.36	6.36	5.36	4.36
	0.36	3.36	nd	nd	nd	nd	5.36	2.36	5.36	5.36	5.36	5.36	5.36	5.36
	3.36	2.36	nd	2.36	nd	3.36	4.36	3.36	4.36	5.36	5.36	4.36	5.36	5.36
	1.36	1.36	nd	nd	nd	nd	5.36	1.36	5.36	4.36	5.36	5.36	5.36	5.36
	3.36	0.36	nd	nd	nd	0.36	7.36	2.36	5.36	3.36	6.36	4.36	6.36	4.36
	3.36	3.36	nd	nd	nd	1.36	5.36	4.36	5.36	4.36	5.36	6.36	4.36	5.36
	0.36	0.36	nd	nd	nd	nd	5.36	4.36	6.36	5.36	6.36	4.36	5.36	4.36
	1.36	2.36	nd	nd	nd	nd	5.36	3.36	6.36	3.36	5.36	5.36	6.36	4.36
	2.36	1.36	nd	nd	nd	1.36	6.36	4.36	8.36	4.36	7.36	4.36	6.36	5.36
	2.36	2.36	nd	nd	nd	nd	5.36	2.36	6.36	3.36	6.36	4.36	6.36	5.36
	2.36	3.36	nd	3.36	nd	3.36	5.36	3.36	6.36	4.36	6.36	5.36	5.36	5.36
	3.36	3.36	nd	nd	nd	2.36	5.36	4.36	6.36	3.36	7.36	4.36	7.36	4.36
	0.36	2.36	0.36	nd	1.36	1.36	5.36	4.36	5.36	5.36	5.36	4.36	5.36	4.36
			nd	nd	nd	nd	5.36	2.36	8.36	3.36	4.36	5.36	6.36	4.36
			nd	nd	nd	nd	5.36	3.36	6.36	5.36	5.36	4.36	6.36	5.36
			nd	nd	nd	nd	4.36	3.36	4.36	3.36	6.36	4.36	6.36	3.36
			nd	nd	nd	1.36	4.36	4.36	5.36	3.36	5.36	2.36	4.36	4.36
			nd	nd	nd	0.36	3.36	4.36	5.36	3.36	5.36	4.36	5.36	5.36
			nd	nd	nd	1.36	6.36	3.36	6.36	4.36	6.36	4.36	6.36	3.36
			nd	nd	nd	nd	5.36	4.36	7.36	4.36	6.36	4.36	5.36	3.36
			nd	2.36	nd	3.36	5.36	5.36	4.36	5.36	6.36	5.36	6.36	5.36
			4.36	3.36	nd	3.36	4.36	2.36	5.36	3.36	4.36	4.36	4.36	2.36
			nd	nd	nd	1.36	5.36	3.36	5.36	4.36	6.36	4.36	6.36	5.36
			nd	0.36	nd	3.36	4.36	5.36	5.36	4.36	6.36	4.36	6.36	4.36
			nd	nd	nd	nd	5.36	4.36	4.36	3.36	6.36	4.36	6.36	4.36
			nd	3.36	nd	2.36	4.36	3.36	5.36	5.36	6.36	5.36	6.36	4.36
			nd	nd	nd	0.36	4.36	3.36	5.36	3.36	6.36	5.36	5.36	6.36
			nd	nd	nd	3.36	5.36	2.36	5.36	3.36	6.36	4.36	6.36	4.36
			nd	2.36	1.36	3.36	5.36	4.36	6.36	4.36	6.36	5.36	5.36	5.36
			nd	nd	nd	nd	4.36	3.36	4.36	4.36	6.36	3.36	5.36	4.36
			nd	nd	nd	nd	6.36	7.36	5.36	4.36	6.36	5.36	7.36	4.36
**median log_{10} MPN/cloacal swab**	**1.86**	**2.36**	**2.36**	**2.36**	**1.36**	**1.72**	**5.36**	**3.36**	**5.36**	**4.36**	**6.36**	**4.36**	**6.36**	**4.36**
**Log reduction**		**-**		**-**		**-**		**2**		**1**		**2**		**2**

[1]median values in log_{10} MPN/g from seeders n = 18; [2]median values in log_{10} MPN/g from sentinels n = 36; "nd" indicates that *C. jejuni* was "not detectable".
