# Supplementary material for: A Complex Competitive Exclusion Culture Reduces Campylobacter jejuni Colonization in Broiler Chickens at Slaughter Age In Vivo
Source: Vet Sci. 2022 Apr 11;9(4):181. doi: 10.3390/vetsci9040181 (PMC9029414; doi:10.3390/vetsci9040181)
Supplement: Supplementary file 1 [file vetsci-09-00181-s001.zip › Supplementary table S4.pdf]

Supplementary Table S4. *C. jejuni* cecal and colon colonization of sentinel broiler chickens at 33 days of age after necropsy.

	**Cecum**		**Colon**	
	**control**	**CE-culture**	**control**	**CE-culture**
	8.36	5.36	7.36	5.36
	7.36	6.36	7.36	6.36
	8.36	6.36	6.36	7.36
	7.36	7.36	9.36	5.36
	7.36	7.36	7.36	7.36
	6.36	6.36	6.36	6.36
	7.36	9.36	6.36	7.36
	8.36	6.36	8.36	5.36
	7.36	7.36	7.36	5.36
	7.36	7.36	7.36	6.36
	7.36	7.36	7.36	8.36
	6.36	6.36	4.36	6.36
	7.36	6.36	6.36	6.36
	7.36	6.36	6.36	5.36
	8.36	6.36	7.36	5.36
	7.36	6.36	6.36	6.36
	7.36	5.36	5.36	4.36
	6.36	8.36	5.36	5.36
	7.36	6.36	7.36	5.36
	8.36	7.36	7.36	7.36
	8.36	5.36	7.36	3.36
	6.36	5.36	6.36	5.36
	6.36	7.36	7.36	5.36
	7.36	6.36	6.36	5.36
	7.36	8.36	6.36	6.36
	8.36	6.36	9.36	6.36
	7.36	7.36	7.36	5.36
	6.36	6.36	6.36	5.36
	9.36	4.36	8.36	5.36
	7.36	7.36	6.36	7.36
	7.36	6.36	7.36	5.36
	8.36	5.36	8.36	6.36
	6.36	6.36	7.36	8.36
	7.36	5.36	5.36	5.36
	8.36	6.36	7.36	4.36
	7.36	7.36	6.36	6.36
**median $\log_{10}$ MPN/g**	**7.36**	**6.36**	**7.36**	**5.36**
**log reduction**	**1**		**2**	
